# Chitin Synthase Is Required for Cuticle Formation and Molting in the Chinese Mitten Crab *Eriocheir sinensis*

**DOI:** 10.3390/ijms26052358

**Published:** 2025-03-06

**Authors:** Ting Zhang, Yuning Hu, Siyu Lu, Yanfei Deng, Huimin Zhang, Yanhua Zhao, Yawen Yu, Hongbin Huang, Jun Zhou, Xuguang Li

**Affiliations:** 1 Key Laboratory of Genetic Breeding and Cultivation for Freshwater Crustacean, Ministry of Agriculture and Rural Affairs, Freshwater Fisheries Research Institute of Jiangsu Province, Nanjing 210017, China; braveting04@163.com (T.Z.); huxiaoao@163.com (Y.H.); lusiyu0801@163.com (S.L.); yf-deng@163.com (Y.D.); huiminzhang_6@163.com (H.Z.); njzhaoyanhua@163.com (Y.Z.); 18851176586@163.com (Y.Y.); nanjingrice@163.com (H.H.); 2 Jiangsu Key Laboratory of Marine Biotechnology, College of Marine Science and Fisheries, Jiangsu Ocean University, Lianyungang 222005, China; 3 Jiangsu Key Laboratory for Aquatic Crustacean Diseases, College of Marine Science and Engineering, Nanjing Normal University, Nanjing 210023, China

**Keywords:** chitin synthase, molting, RNA interference, *Eriocheir sinensis*

## Abstract

Chitin synthase is an essential enzyme of the chitin synthesis pathway during molting. In this study, we identified and characterized a chitin synthase (*EsCHS*) gene in the Chinese mitten crab, *Eriocheir sinensis*. The spatio-temporal expression and functional role of *EsCHS* were investigated. The open reading frame of *EsCHS* was 4725 bp long and encoded 1574 amino acid residues that contained the typical domain structure of the glycosyltransferase family 2. Phylogenetic analysis revealed that *EsCHS* belongs to the group I chitin synthase family. The expression of *EsCHS* was found in regenerative limbs, the cuticle and the intestines. During the molting cycle, *EsCHS* began to increase in the pre-molt stage and reached a significant peak in the post-molt stage. The knockdown of *EsCHS* resulted in the significant downregulation of chitin biosynthesis pathway genes, including *TRE*, *HK*, *G6PI*, *PAGM* and *UAP*. Moreover, the long-term RNAi of *EsCHS* resulted in thinning procuticles, abnormal molting and high mortality, suggesting that *EsCHS* is indispensable for the formation of chitin in the cuticle during molting. In conclusion, *EsCHS* is involved in the chitin biosynthesis pathway and plays an important role in molting in *E. sinensis*. These findings highlight the potential of incorporating *EsCHS* into selective breeding programs to optimize molting regulation and improve growth performance in crustacean aquaculture.

## 1. Introduction

Chitin is a linear polymer composed of N-acetylglucosamine units connected by β-1, 4-glycoside bonds and is the second most abundant biopolymer in nature. In crustaceans, chitin is the principal structural component of the exoskeleton, peritrophic matrix (PM) and trachea. Chitin enhances the mechanical strength of the insect exoskeleton and protects intestinal health. However, as the size of crustaceans increases, crustaceans periodically have to discard their rigid exoskeleton and synthesize a new exoskeleton to match their new body size during molting [1]. Chitin biosynthesis is a sophisticated process that encompasses a series of catalytic reactions. Several genes encoding the enzymes related to the upstream and downstream parts of this pathway play a vital and indispensable role in this process, including trehalase (*TRE*), hexokinase (*HK*), glucose-6-phosphate isomerase (*G6PI*), glutamine-fructose-6-phosphate aminotransferase (*GFAT*), glucosamine-6-phosphate-n-acetyltransferase (*GNPNA*), phosphoacetylglucosamine mutase (*PAGM*), uridine diphosphate-N-acetylglucosamine pyrophosphorylase (*UAP*) and chitin synthase (*CHS*) [2]. These genes are not only key elements in the chitin biosynthesis pathway but also potential targets for in-depth research on the regulatory mechanisms of this process. Chitin synthase is an indispensable enzyme that catalyzes the last step of the reaction in chitin biosynthesis. Chitin synthase (CHS) belongs to family 2 of β-glycosyltransferases (GT2), which also includes cellulose synthases. CHSs have been extensively studied in insects [3]. Most insects contain two kinds of CHSs, CHS-1 and CHS-2 [4]. Several studies have shown that these two genes are involved in the synthesis of chitin in different insect body parts. CHS-1 is mainly involved in chitin synthesis in the epidermis and trachea, while CHS-2 is highly expressed only in insect midgut epithelial cells and is responsible for synthesizing chitin in the peritrophic membrane [5,6].

In contrast to insects, the role of CHS in chitin synthesis is rarely reported in crustaceans. Rocha first identified the partial sequence of a gene encoding crustacean chitin synthase in *Penaeus vannamei*, and Wang cloned the complete cDNA of chitin synthase in *Macrobrachium nipponense* [7,8]. Since then, with the development of the transcriptome and whole genome, more genes that encode crustacean chitin synthases have been discovered in *Pandalopsis japonica*, *Penaeus japonicus* and *Macrophthalmus japonicus* [9,10,11]. Based on relative sequence differences, chitin synthases have been grouped into two classes, class CHS-A and class CHS-B enzymes, in planktonic crustaceans. Meanwhile, most decapods seem to have one gene copy for each enzyme [12]. In *Lepeophtheirus salmonis*, *LsCHS1* is expressed in the antenna, intestines and feet in different life stages, while *LsCHS2* is most highly expressed in the intestines of adult lice. The knockdown of *LsCHS1* in pre-adult I lice resulted in lethal phenotypes with cuticle deformation and the deformation of ovaries and oocytes in adult lice. The knockdown of *LsCHS2* in adult female *L. salmonis* affected digestion, damaged the gut microvilli, reduced muscular tissues around the gut and affected offspring [13,14]. During periodic molting, CHSs were mainly highly expressed in the post-molting stage. Eyestalk ablation and 20 hydroxyecdysone injection increased the expression level of CHS, suggesting that the expression of CHS may be involved in molting [7,9]. However, it is unclear whether chitin synthases in crustaceans share functional roles in molting.

The Chinese mitten crab (*Eriocheir sinensis*) is one of the most important aquaculture species in China. In recent years, with the expansion of large-scale aquaculture and the increase in aquaculture density, the mortality of molting has increased, and the growth rate of molting has decreased, thus becoming an important bottleneck restricting the development of *E. sinensis* aquaculture. Periodic molting occurs throughout the life history of *E. sinensis* and is essential for metamorphosis, growth, regeneration and reproduction [15]. Studies on molting, especially cuticle degradation and synthesis, are valuable for *E. sinensis* eco-aquaculture. A series of enzymes, including chitinase and chitin deacetylase, is involved in the hydrolysis and synthesis of chitin during molting in *E. sinensis* [16,17]. However, little is known regarding the chitin synthase gene (*EsCHS*) of *E. sinensis*. To gain a deeper understanding of the biological role of *EsCHS* in *E. sinensis*, we evaluated the expression of the *EsCH* gene across the molting stages of *E. sinensis* as well as the expression of the *EsCHS* gene in various tissues. In addition, a loss-of-function analysis of the *EsCHS* gene via dsRNA-induced silencing was performed.

## 2. Results

### 2.1. Identification and Characterization of EsCHS

**Figure 1:**
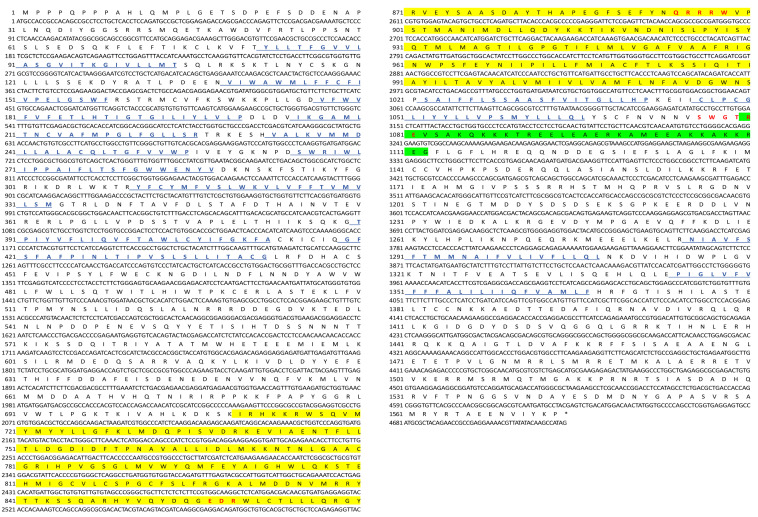
The nucleotide and deduced amino acid sequences of *EsCHS*. Blue font and underline: transmembrane region; yellow area: Chitin_synth_2 domain; red font: unique motifs; green area: coiled coil region.

**Figure 2:**
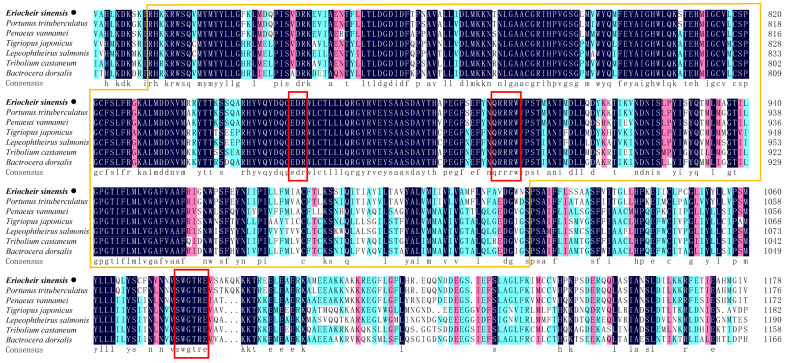
The multiple alignment of the deduced amino acids of CHSs among *E. sinensis* and other different species. The yellow box area: Chitin_synth_2 domain; red box area: unique motifs. *EsCHS* from *E. sinensis* is highlighted in bold and black dots.

**Figure 3:**
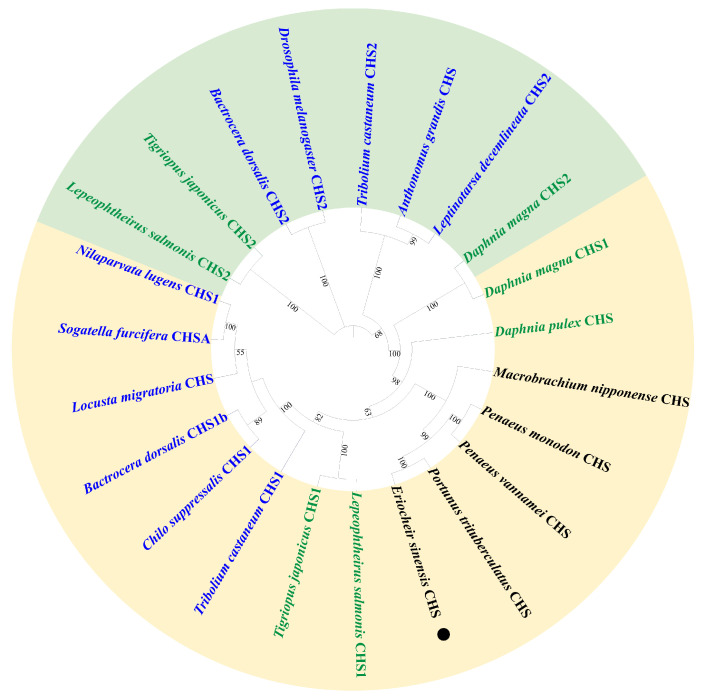
Phylogenetic analysis of organism chitin synthase (CHS) based on catalytic domain amino acid sequences. Bootstrap analysis of 1000 replications was carried out on trees inferred from Neighbor-joining method. *EsCHS* from *E. sinensis* is highlighted with black dots. Yellow area: CHS1; green area: CHS2; black font: crustacean; green font: copepoda and cladocera; blue font: insect.

The cDNA sequences encoding putative chitin synthase were identified in *E. sinensis* transcriptome data and designated as *EsCHS* (GenBank accession number: LOC126999678). The ORF of *EsCHS* ranged around 4725 bp long with the putative proteins of 1574 amino acid residues. The theoretical molecular mass of *EsCHS* was about 180.63 kDa with a predicted isoelectric point of 6.74. Domain analysis indicated that *EsCHS* contained three conserved domains: the A, B, and C domains. Domain A is located at the N end and contains nine transmembrane helices, domain B is the active center of CHS, and domain C is located in terminal C which contains a coiled coil region, a highly conserved unique motif, SWGTRE, and four transmembrane helices. The B domain is highly conserved and contains two unique motifs, EDR and QRRRW, that are essential for the catalytic mechanism, since they are involved in the protonation of the substrate (Figure 1). The multiple sequence alignment of the deduced protein sequences showed that *EsCHS* shared 92.04% and 58.62% identity with the CHSs of *Portunus trituberculatus* and *Tigriopus japonicus* from other crustaceans, respectively (Figure 2). Phylogenetic analysis based on domain architecture showed that arthropod CHSs were divided into two major groups, and the sequences of *EsCHS* were clustered in a separate clade in crustacean group, closely related to *PtCHS*, *PvCHS*, *PmCHS* and *MnCHS*, as well as other crustaceans (Figure 3).

### 2.2. Expression of EsCHS in Different Tissues

**Figure 4:**
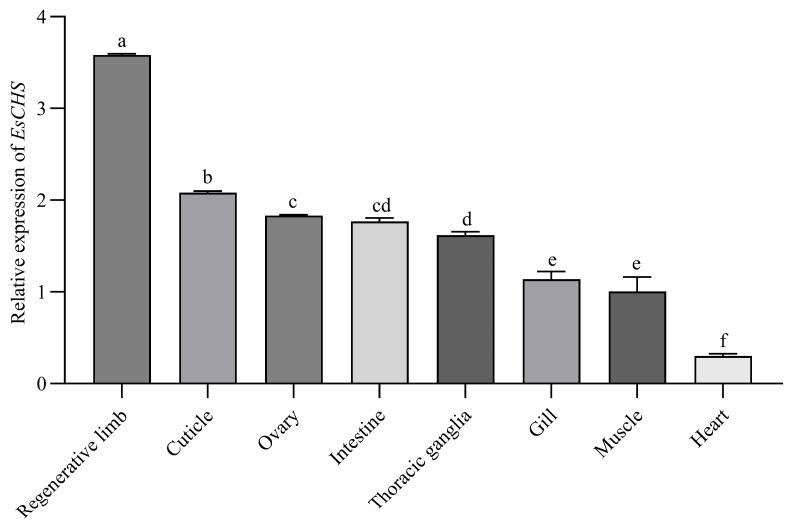
Spatial expression of *EsCHS* gene in eight different tissues of *E. sinensis*. Gene expression levels were measured by quantitative real-time PCR with β-actin as internal control and transformed using log10 (relative expression + 1) to stabilize variance and improve normality. Values are means ± SE from three independent measurements (*n =* 3). Different letters indicate significant differences according to Duncan’s test (*p* < 0.05).

*EsCHS* expression levels in different tissues of *E. sinensis* were determined by qPCR. The results showed that *EsCHS* was widely expressed in chitinous tissues. *EsCHS* expression levels were high in the regenerative limb and cuticle, and its expression levels in the ovaries, intestines, thoracic ganglia and gills were relatively low (Figure 4).

### 2.3. Expression of EsCHS in Molting Cycle

**Figure 5:**
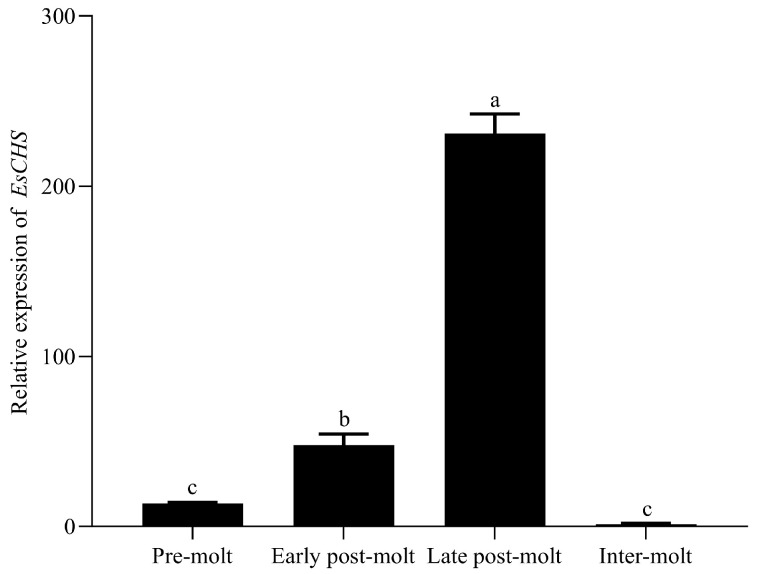
Expression levels of *EsCHS* in four different molting stages in cuticle of *E. sinensis*. Gene expression levels were measured by quantitative real-time PCR with β-actin as internal control. Values are means ± SE from three independent measurements (*n =* 3). Different letters indicate significant differences according to Duncan’s test (*p* < 0.05).

According to the qPCR results, the cuticle-relative expression trends in *EsCHS* increased in the pre-molt stage, reached a peak in the late post-molt stage and then fell back in the inter-molt stage (Figure 5).

### 2.4. Expression of EsCHS and Chitin Biosynthesis-Related Genes After RNAi

**Figure 6:**
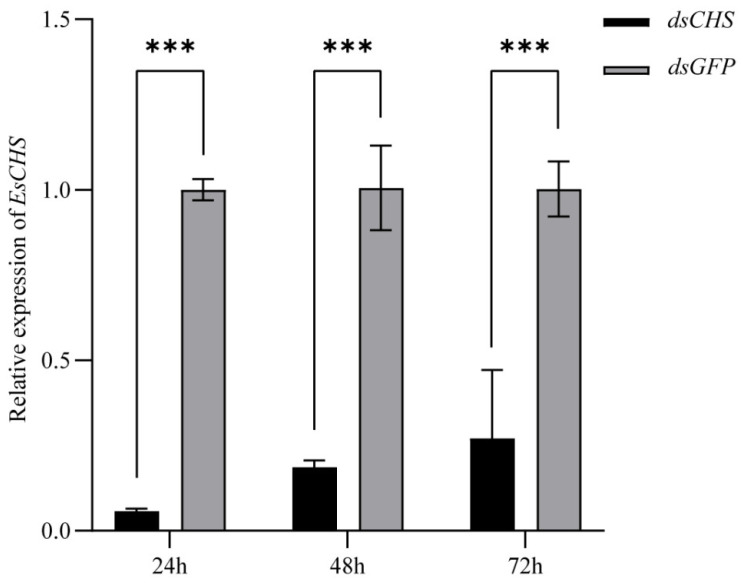
Effects of *dsCHS* on cuticle of *E. sinensis* after dsRNA treatment. Values are means ± SE from three independent measurements (*n =* 3). Three asterisks indicate extremely significant difference according to Tukey’s test (*p* <  0.001).

**Figure 7:**
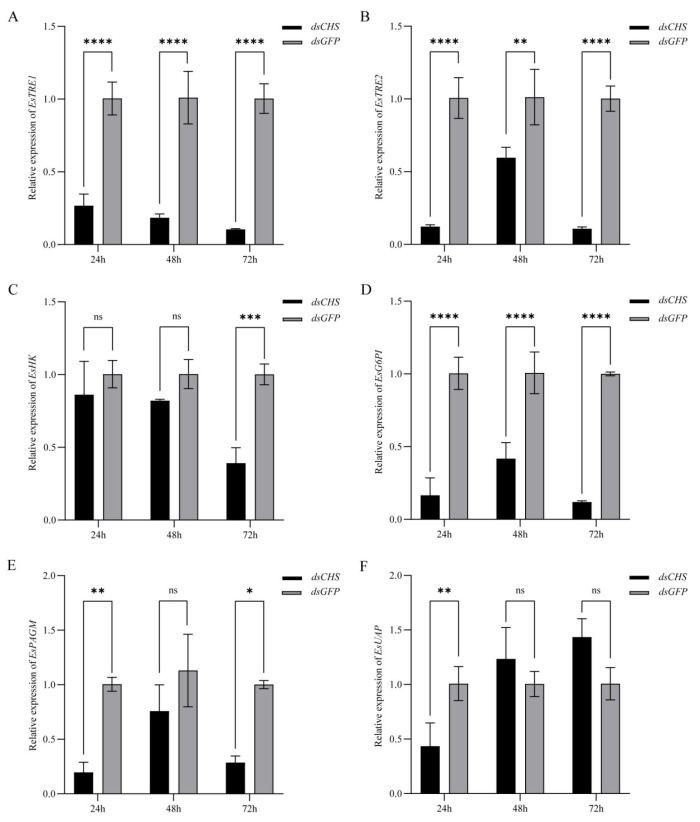
Expression of genes involved in chitin synthesis pathway in cuticle of *E. sinensis* after dsRNA treatment. (**A**): *TRE1*; (**B**): *TRE2*; (**C**): *HK*; (**D**): *G6PI*; (**E**): *PAGM*; (**F**): *UAP*. Values are means ± SE from three independent measurements (*n =* 3). Different number of asterisks indicates significant differences according to Tukey’s test (besides “ns” representing *p* > 0.05).

The results of qPCR showed that the relative expression level in the cuticle of *EsCHS* was significantly decreased at 24 h, 48 h and 72 h after *dsCHS* injection, which suggests that dsRNA successfully inhibited the expression in the cuticle of the target gene *EsCHS* (Figure 6). The knockdown of *EsCHS* resulted in the expression levels of all five genes in the chitin biosynthesis pathway becoming significantly inhibited (*p* < 0.05). Among them, *TRE1* exhibited a progressive downregulation throughout the experimental period. *TRE2*, *G6PI* and *PAGM* displayed transient upregulation at 48 h followed by a subsequent decline. *HK* maintained stable expression levels during the early stages (24–48 h) with significant downregulation only observed at 72 h. In contrast, *UAP* showed an acute but transient suppression at 24 h, followed by recovery to baseline levels with no statistically significant difference in the subsequent time points (*p* > 0.05) (Figure 7).

### 2.5. Functional Impact of EsCHS Knockdown

**Figure 8:**
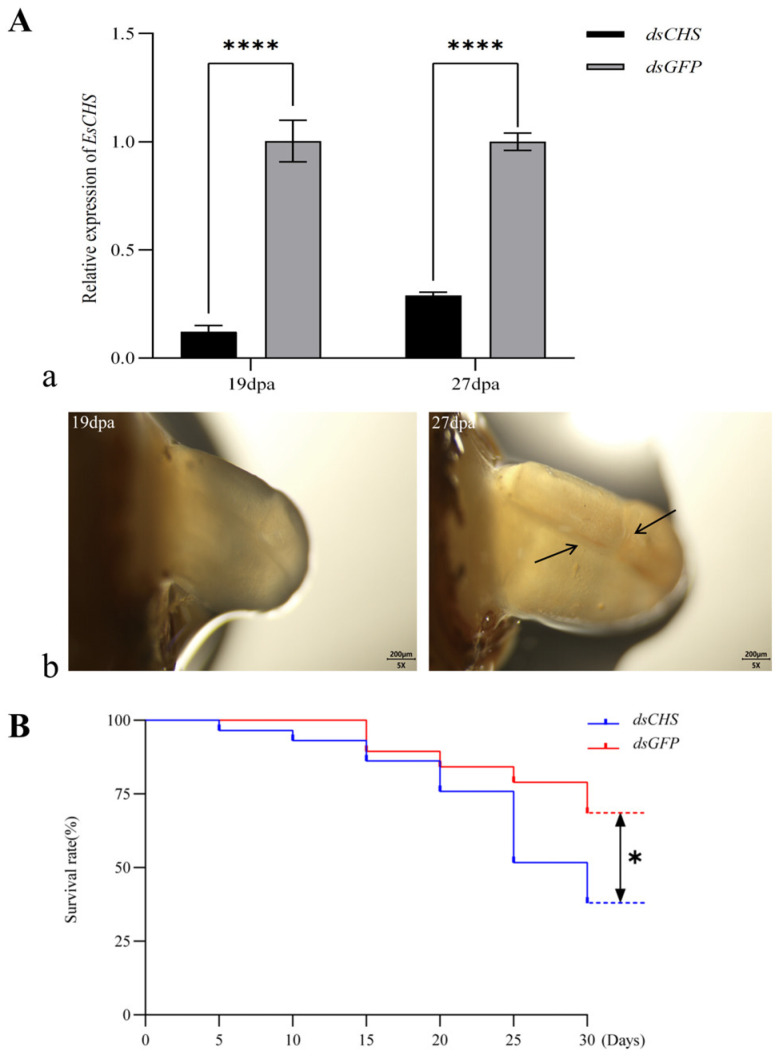
Effects of *dsCHS* on *E. sinensis* and changes in survival rate within experimental period. (**A**) Relative expression and phenotypic changes during limb regeneration of *E. sinensis*. (**a**) Expression of genes at basal growth stage (19 dpa) and proecdysial growth stage (27 dpa) in *E. sinensis* after dsRNA treatment. (**b**) Morphology of regenerative limbs of *dsCHS* at basal growth stage (19 dpa) and proecdysial growth stage (27 dpa) under dissecting microscope; arrow is directed at blastema fold that emerges for first time, suggesting that regenerative appendages were on verge of entering proecdysial growth stage. (**B**) Survival status of *E. sinensis* under influence of dsRNA during experimental period. Values are means ± SE from three independent measurements. Four asterisks indicate ultra-high significant difference (*p* < 0.0001), and one asterisk indicates significant difference according to Tukey’s test (*p* < 0.05).

The regenerative limb morphology of *E. sinensis* was continuously observed under a dissection microscope following long-term RNA interference (RNAi) targeting *EsCHS* (*dsCHS*). During the RNAi process, gene knockdown efficiency was monitored via qPCR, which demonstrated a sustained knockdown efficiency exceeding 70% (Figure 8A(a)). As shown in Figure 8A(b), by day 27 post-amputation (dpa), clear blastema folds were observed in the limb buds, indicating that the regenerative appendages were approaching the proecdysial growth stage. Meanwhile, the survival curves revealed a marked divergence between the experimental and control groups in mortality rates starting from days 25–30. The molting mortality rate was 56.67% in the RNAi-treated group, compared to 20% in the control group (Figure 8B).

**Figure 9:**
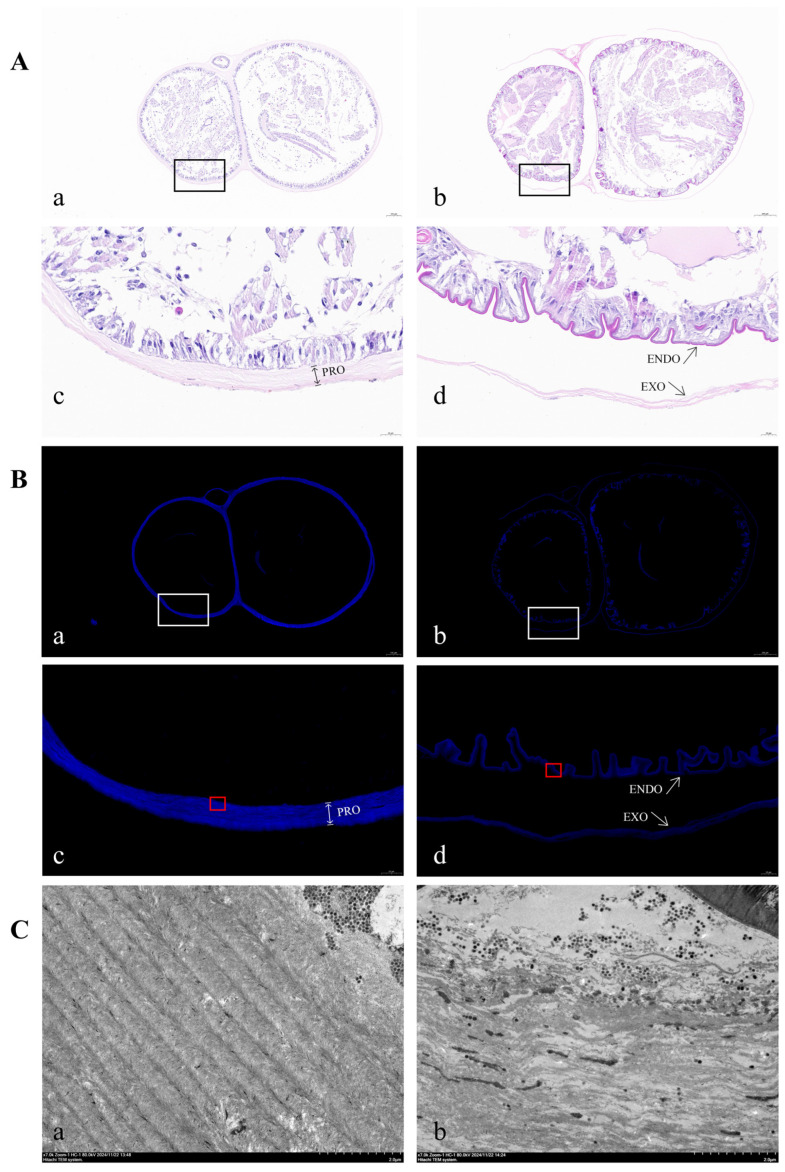
H&E staining, FB28 staining and ultrastructure of regenerative limbs at proecdysial growth stage (27 dpa). (**A**) Cross-sections of regenerative limbs stained with H&E. Control group, represented by “a”, was injected with *dsGFP*, while experimental group, labeled as “b”, was injected with *dsCHS*; “c” and “d” are detailed images of “a” and “b” regions highlighted by black box magnified × 6.8, respectively. (**B**) Cross-sections of regenerative limbs stained with FB28. Control group, represented by “a”, was injected with *dsGFP*, while experimental group, labeled as “b”, was injected with *dsCHS*; “c” and “d” are detailed images of “a” and “b” regions highlighted by white box magnified × 6.8, respectively. Red box region: part of the endocuticle. (**C**) Cross-sections of regenerative limbs with ultrastructure (TEM) (×7.0k). Control group, represented by “a”, is magnified view within red box region in panel B-c and was injected with *dsGFP*. Experimental group, labeled as “b”, is magnified view within red-bordered region in panel B-d. Procuticle is composed of endocuticle and exocuticle. EXO: exocuticle; ENDO: endocuticle; PRO: procuticle.

**Figure 10:**
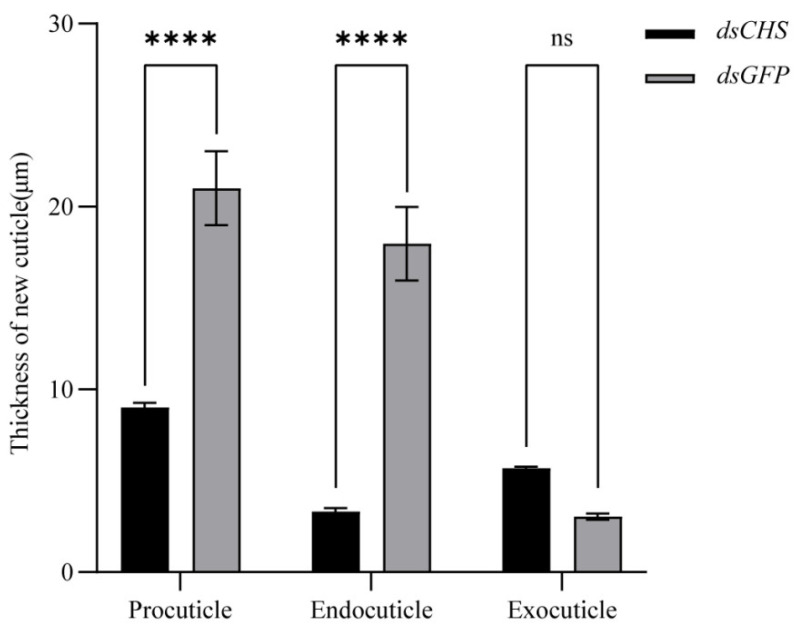
Procuticle thickness measurement in regenerative limb of *E. sinensis* at proecdysial growth stage (27 dpa) after dsRNA treatment. Procuticle = Endocuticle + Exocuticle. Values are means ± SE from three independent measurements. Four asterisks indicate ultra-highly significant difference (*p* < 0.0001), and “ns” indicates significant difference according to Tukey’s test (*p* < 0.05).

Hematoxylin–eosin (HE) staining, fluorescent brightener 28 (FB28) staining and transmission electron microscopy (TEM) were employed to analyze the ultrastructure of the newly formed cuticle in the regenerative limb. The results showed that the cuticle was intact in the control group, whereas the separation and invagination of the cuticle were observed in the RNAi group (Figure 9A,B). A TEM analysis of the endocuticle layers revealed that the control group exhibited a well-organized arrangement of horizontal chitin lamellae, while the RNAi group showed a disorganized and loose structure (Figure 9C). Further measurements of procuticle thickness in the regenerative limbs at the proecdysial growth stage (27 dpa) following dsRNA treatment revealed that the control group exhibited a dense multilayered structure with an average procuticle thickness of 21 μm. In contrast, the RNAi group exhibited an average procuticle thickness of 9 μm (Figure 10).

## 3. Discussion

Chitin synthases are the crucial enzymes of the chitin synthesis pathway and play vital roles in molting. Although many reports have extensively highlighted the importance of chitin synthesis in insects, little is known in crustaceans [3]. It has only been reported in *M. nipponense*, *P. japonica*, *P. vannamei*, *P. japonicus* and *M. japonicus* at present [2]. In the current study, we identified a novel chitin synthase gene from *E. sinensis* for the first time. The expression patterns of *EsCHS* in various tissues and different molting stages were analyzed. Furthermore, we investigated the physiological function of chitin synthase by RNAi in *E. sinensis*. Sequence alignment analysis revealed that the *EsCHS* catalytic domain contains the highly conserved chitin synthase signature motifs EDR, QRRRW and SWGTKG. The site-directed mutagenesis of the EDR and QRRRW motifs results in the loss of activity, suggesting that these two motifs are essential for CHS activity. SWGTRE motifs face the extracellular environment and play an important role in chitin translocation. The distribution and conserved number of these 13 transmembrane helices (TMHs) in *EsCHS* allow the central catalytic domain to face the cytoplasm, where the UDP-*N*-acetylglucosamine (UDP-GlcNAc) substrate is accessible [18]. The phylogenetic analysis indicated that CHS from *E. sinensis* belongs to the CHS1 group. Generally, insects, cladocera and copepods have two CHS genes. Interestingly, there is just one CHS gene in decapoda according to several studies [19]. This study also supports the finding that crustaceans only contain one CHS.

Tissue-specific expression analyses revealed that *EsCHS* was predominately expressed in the regenerative limb and cuticle. This is consistent with the fact that CHS is responsible for chitin biosynthesis [20,21]. Similar results have been observed in *P. japonicus* and *P. Japonica*. The growth of crustaceans is accompanied by periodic molting, during which crustaceans expand their exoskeleton to adapt to internal growth. During this process, crustaceans need to significantly increase exoskeletal chitin levels to form a new cuticle [22]. Our results indicated that the trends in the expression of *EsCHS* were periodically repeated in each molting cycle. The expression of *EsCHS* peaked after molting, declined during each inter-molting phase and then increased again before the next molt. Similar phenomena have also been observed for the transcript patterns of CHS in *M. nipponense*, *P. japonica* and *P. vannamei* [7,8,9]. The significant post-molt rise in the molting cycle was probably related to the requirement for chitin synthesis during molting.

Gene silencing through dsRNA injection was successfully used to study the functions of essential genes in crustaceans. The results of qPCR showed that the relative expression levels of *EsCHS* were significantly decreased after dsRNA injection, suggesting that dsRNA successfully inhibited the expression of target genes. In this study, when *EsCHS* was inhibited by RNAi, the relative expression levels of these genes were decreased significantly. This result is consistent with previous findings in *L. salmonis* and *Aedes. albopictus* [13,23]. Notably, *EsCHS* inhibition elicited fluctuating expression profiles of *HK* and *UAP*, potentially indicating the existence of compensatory regulatory circuits or feedback mechanisms. These oscillatory patterns may result from the complex interplay between synergistic and antagonistic regulatory elements within the chitin metabolic pathway. Therefore, silencing CHS expression directly leads to impaired chitin synthesis by inhibiting the chitin pathway genes.

Chitin is the major scaffolding component of the crustacean cuticle. The precise control of chitin synthesis is mandatory to ensure the correct chitin assembly and proper function of cuticle structures. Thus, the evaluation of chitin-deficient phenotypes is key to our understanding of the function of the enzymes involved in chitin metabolism. The hard exoskeleton covers the whole body of *E. sinensis*, and it is not easy to observe the newly synthesized cuticle under the exoskeleton during molting. It was reported that autotomy and regeneration happened at the base segment of the limbs immediately after injury. Subsequently, the blastema cells proliferate to secrete a multilayer cuticular structure called limb buds in the basal growth stage and form a functional new limb after the subsequent molting [24,25]. In this complex process, the regenerated limbs are exposed, which provides us with the opportunity to observe the morphological structure of the cuticle. The formation of the blastema fold suggests that the newly formed limb bud folds and wraps within a cuticular sac prior to molting. In this study, by day 27 post-amputation (dpa), a clear blastema fold was observed in the limb buds, indicating that the regenerative appendages were approaching the proecdysial growth stage. FB28 is a fluorescent brightener widely used in the topological localization of chitin, commonly used in fungal cell wall and insect cuticle fluorescent microscopy [26,27]. Here, we use FB28 for the chitin-specific staining of the regenerative limb cuticle in *E. sinensis.* The fluorescence obtained from FB28 reflects the distribution and thickness of chitin in the procuticle, indicating that the FB28 staining method is a reliable technique for chitin-specific staining in *E. sinensis* and may be applied to other crustaceans.

Long-term RNAi caused procuticle deformation and abnormal molting, and the mortality rates of *E. sinensis* were also increased extremely significantly compared to the control group. Similar results have been observed in other crustaceans, in which the silencing of CHS expression by in vivo RNAi caused phenotypic defects in molting and resulted in the mortality of the injected crustaceans [19]. In *L. salmonis*, silencing the expression of *LsCHS1* resulted in abnormal molting and a reduction in chitin in the exoskeleton, while the silencing of *LsCHS2* did not affect ecdysis or exoskeleton chitin formation [14]. In *M. japonicus*, suppressing the expression of *MjCHS* led to decreased survival and exoskeleton surface profile changes. The knockout of *CsCHS1* by CRISPR/Cas9 genome editing severely lowered the hatching rate, larval survivorship, pupation rate and eclosion rate in *Chilo suppressalis* [28]. In addition, chitin synthase expression was significantly downregulated, and aberrant cuticle forms, abnormal procuticle depositions and abortive molting were observed in *T. japonicus* exposed to a chitin synthesis inhibitor [29,30]. These suggested that the silencing of CHS affects the synthesis of chitin by interfering with the transcription levels of crucial chitin-synthesizing enzymes, thus resulting in cuticle malformation and abortive molting. Molting failure in aquaculture, particularly under high-density farming, is likely driven by stress-induced chitin metabolism disruption. *EsCHS* knockdown-induced mortality and defects underscore its critical role in chitin biosynthesis, and potential applications lie in positioning it as a potential biomarker for monitoring the readiness of molting or as a target for dietary supplementation to enhance the integrity of the procuticle. In addition, the severe mortality and molting abnormalities in *EsCHS*-silenced crabs imply that the gene has an important role in survival. This finding provides support for incorporating *EsCHS* into selective breeding programs, aiming to regulate molting behavior and enhance growth.

In conclusion, we identified chitin synthase *EsCHS* in *E. sinensis*, and the expression patterns of *EsCHS* in various tissues and different molting stages were examined. The RNAi of *EsCHS* caused the abnormal ultrastructure of the cuticle, blocked molting and led to high mortality. Overall, our study revealed that chitin synthase can affect cuticle chitin synthesis by regulating chitin metabolic pathway-related genes and is involved in the structure of the cuticle in *E. sinensis*, playing an important role in molting.

## 4. Materials and Methods

### 4.1. Specimen and Culture Conditions

Following assessments of health status, integrity and size specifications, Chinese mitten crabs (*n =* 200; 20 ± 0.5 g wet weight) were collected from the Genetic and Breeding Center, Freshwater fisheries research institute of Jiangsu province, China. The crabs were acclimatized and cultured for one week in aquaculture tanks in a filtered, aerated freshwater system before the beginning of this experiment. Based on the developmental stage of juvenile crabs, a formulated feed with a protein level of 35% and a fat level of 12% was used for satiation feeding. The crabs were fed twice daily to satiation, and any uneaten feed was removed from the tanks two hours after each feeding session. Dead crabs were promptly removed to maintain water quality. During the experimental period, continuous aeration was provided to the culture water, ensuring dissolved oxygen levels remained above 7.5 mg/L at room temperature. Approximately one-third of the water was replaced every two days to maintain optimal water conditions. The samples of various tissues including the regenerative limb, cuticle, muscle, gill, thoracic ganglia, heart, intestine and ovary in *E. sinensis* were collected to examine the tissue-specific expression profiles of *EsCHS*. The crabs at different stages were divided into four groups, inter-molt (C), pre-molt (D), early post-molt (A) and late post-molt (B), with each group having at least three crabs based on the morphological changes in setogenesis during the molting cycle. The cuticle from each group of crabs were dissected and collected for the examination of the molting-specific expression profiles of *EsCHS*. All the samples were immediately snap-frozen in liquid nitrogen and stored at −80 °C until use.

### 4.2. RNA Extraction and Sequence Verification

Total RNA was extracted from various tissues and different molting stages and developmental stages using TaKaRa MiniBEST Universal RNA Extraction Kit (TaKaRa, Dalian, China). The extracted total RNA was evaluated using agarose gel electrophoresis, and its concentration was measured by a NanoDrop™ 2000 spectrophotometer (NanoDrop Products, Wilmington, DE, USA) and immediately stored at −80 °C for storage. The extraction RNA was treated with the PrimeScript RT Master Mix (Perfect Real Time) (TaKaRa, Dalian, China) to Synthetic first-strand cDNA and then stored at −20 °C for storage. For qPCR expression analysis, total RNA was reverse-transcribed using the TB Green^®^ Premix Ex Taq™ II (Tli RNaseH Plus) (TaKaRa, Dalian, China). Based on the molting transcriptome of the cuticle of *E. sinensis*, further screening was conducted to obtain the gene sequence of *EsCHS* in *E. sinensis*. To confirm the *EsCHS* cDNA sequence, the corresponding pairs of gene-specific primers (Table S1) were designed to amplify the *EsCHS* ORF. The PCR mixtures (25 µL) contained 12.5 µL 2 × PCR mix, 1 µL of the first-strand cDNA, 1 µL forward primer (10µM), and 1 µL reverse primer (10µM), and the rest was filled by DEPC-treated water. The PCR program was under the following conditions: 94 °C for 5 min, 35 cycles (94 °C for 30s, 60 °C for 45s and extension at 72 °C for 2 min) and 72 °C for 5 min. The PCR products were then gel-purified and sequenced by Sangon Biotech (Shanghai, China).

### 4.3. Bioinformatical Analysis

The cDNA sequence and deduced amino acid sequence of *EsCHS* were analyzed using the online program BLAST (http://blast.ncbi.nlm.nih.gov/Blast.cgi, accessed on 17 July 2023) and the Expert Protein Analysis System (https://www.expasy.org/, accessed on 21 August 2023). Multiple domains were identified from the SMART tool (http://smart.embl-heidelberg.de/, accessed on 21 August 2023). The theoretical parameters of the deduced proteins were obtained using online software ExPASy Proteomics Server (https://web.expasy.org/protscale/, accessed on 21 August 2023). To perform a phylogenetic analysis of CHSs, the deduced proteins including those of the crustaceans, insects and other representative organisms were obtained from the NCBI database (Table S2). A phylogenetic tree was constructed based on the catalytic domain using Molecular Evolutionary Genetics Analysis (MEGA 7.0).

### 4.4. Expressional Analysis of EsCHS by Quantitative Real-Time PCR

Specific primers for qPCR were designed using online software Primer 3 Web (http://primer3.ut.ee/, accessed on 5 September 2024) (Table S2) and synthesized by Sangon Biotech (Shanghai, China). The PCRs were performed on a 96-well microtiter plate by using 2 × SYBR Premix *Ex Taq* kit (Perfect Real Time, Takara, Dalian, China). The reaction system comprised a 2 μL cDNA template, 12.5 μL TB Green™ Premix ExTaq™ (Takara, Dalian, China), 1 μL forward primer, 1 μL reverse primer and 8.5 μL DEPC-treated water. Non-template reactions were maintained as negative controls. The best reference gene for stage- and tissue-specific expression profile analyses was that encoding β-actin. The PCR was as follows: 95 °C for 2 min for 1 cycle, followed by 40 cycles of 95 °C for 15 s and then 60 °C for 1 min. Three biological replicates and three technical replicates were run for qPCR analysis. The relative expression for each sample was calculated using the 2^−ΔΔCt^ method.

### 4.5. Function Analysis of EsCHS by RNA Interference

#### 4.5.1. Short-Term RNA Interference

Double-stranded RNA targeting *EsCHS* was prepared with the TranscriptAid T7 High Yield Transcription Kit (Thermo Fisher Scientific, Waltham, MA, USA) based on the manufacturer’s protocol. Forward and reverse primers containing a T7 RNA Polymerase promoter were designed to amplify 493 bp and 467 bp for *EsCHS* and the green fluorescent protein (*GFP*) gene, respectively. pGEM-T Easy Vector (Promega, Madison, WI, USA) plasmids carrying the gene fragments were used as the template to generate corresponding dsRNA, which was then suspended in DEPC-treated water. The integrity and quantity of dsRNAs were determined using a spectrophotometer with Nanodrop 2000 (Thermo Fisher Scientific, Waltham, MA, USA) and agarose gel electrophoresis. The concentration of dsRNA was adjusted to 5 μg/g per body weight, and the sample was stored at −20 °C until use. *GFP* was used as a negative control for the non-specific effects of dsRNA. Using a manual microinjector (NARISHIGE, Tokyo, Japan), juvenile crabs were injected with dsRNA and *dsGFP* through the base of the third appendage. After the injection, the crabs were kept for a few hours to recover and then returned to the aquaculture system.

For the expression of chitin synthesis-related genes, the inter-molt crabs were separated into two groups, treatment group injected with the dsRNA of *EsCHS* (*n =* 15) and a control group injected with the exogenous dsRNA of *dsGFP* (*n =* 15). After dsRNA was injected into the samples of *E. sinensis* in the inter-molt (C) stage, the relative expression levels in the cuticle of *EsCHS* and chitin synthesis-related genes were analyzed at 24 h, 48 h and 72 h. Through NCBI and the related literature, the genes of chitin synthesis from *E. sinensis* were selected, including *TRE1* (NCBI: XM_050884128.1), *TRE2* (NCBI: XM_050844968.1), *HK* (NCBI: XM_050875290.1), *G6PI* (NCBI: XM_050870315.1), *PAGM* (NCBI: XM_050878423.1) and *UAP* (NCBI: XM_050880871.1).

#### 4.5.2. Long-Term RNA Interference

The third and fourth appendages on the right side of crabs in the inter-molt (C) stage were treated so that the crabs would self-cut (target limbs were gently gripped with forceps, prompting the crab to autonomously shed the appendage without direct surgical incision). The crabs with broken limbs were separated into two groups, a control group injected with the exogenous dsRNA of *dsGFP* (*n =* 30) and a treatment group injected with the dsRNA of *EsCHS* (*n =* 30) every other day. The regenerative limbs that were selected during the pre-molting stage were sliced for the observation of the morphological structure of the cuticle. The abnormal molting and survival rate of other *E. sinensis* were recorded within the 30-day experimental cycle.

Hematoxylin–eosin staining: As described above, the newly formed cuticle was dissected to observe the morphological ultrastructure. Prior to the histological analysis of the cuticle structure in regenerative limbs, excised tissues were fixed overnight in 4% paraformaldehyde (PFA) solution at 4 °C. After 27 d of RNAi, the cuticle of the left third regenerative limb of *E. sinensis* was serially sectioned at a thickness of 4 μm using an RM2016 paraffin microtome and placed onto glass slides. Following overnight exposure at 37 °C, the slides were dewaxed in xylene for 20 min, rehydrated through ethanol gradients and washed twice in DEPC-treated water. For hematoxylin and eosin staining, tissues were stained with hematoxylin for 15 s and eosin for 4 s. Subsequently, the slides underwent treatment with ethanol, followed by two immersions in xylene. Finally, the slides were sealed with neutral gum. The staining results were observed under a Leica microscope for morphological analysis.

Fluorescent brightener 28 chitin staining: To compare the chitin content and distribution in the cuticle of the regenerative limb, paraffin-embedded serial sections were subjected to dewaxing. The sections were stained with fluorescent brightener 28 (FB28) (Sigma-Aldrich, Shanghai, China) for 10 s, washed three times with water for 1 min each time, counterstained with propidium iodide for 10 s and washed three times with water for 1 min each time, and excess water was removed from the air-dried slide. An antifluorescent stain was added dropwise for sealing, and samples were observed under a fluorescence microscope.

Transmission electron microscope analysis: The cuticle of the left fourth regenerative limb was fixed with 3% glutaraldehyde (Solarbio, Beijing, China) for 48 h at 4 °C. The cuticle was then rinsed thrice with PBS followed by post-fixation in 1% osmium tetroxide for 3 h at 4 °C. Subsequently, the cuticle was washed several times and dehydrated in a 10% step-graded acetone series from 50% to 100%. The cuticle was embedded in Epon 812 at room temperature for 2 h and then trimmed to prepare ultrathin sections. Ultrathin sections of the cuticle were stained with 4% uranylacetate and observed using a JEM-1200EX transmission electron microscope (JEOL, Tokyo, Japan).

### 4.6. Statistical Analyses

In this study, all data were analyzed using a one-way analysis of variance (ANOVA) and are shown as the mean ± standard error (SE) of three biological replicates. Data on developmental and tissue expression patterns were analyzed using Duncan’s test. In Duncan’s test, different letters indicate a significant difference (*p* < 0.05). Other data were analyzed using Tukey’s test. In Tukey’s test, a double asterisk indicates an extremely significant difference in mRNA levels (*p* < 0.01), and one asterisk indicates a significant difference (*p* < 0.05).

## Data Availability

The raw data supporting the conclusions of this article will be made available by the authors on request.

## Supplementary Materials

The following supporting information can be downloaded at https://www.mdpi.com/article/10.3390/ijms26052358/s1.
