# Supplementary material for: Chitin Synthase Is Required for Cuticle Formation and Molting in the Chinese Mitten Crab Eriocheir sinensis
Source: Int J Mol Sci. 2025 Mar 6;26(5):2358. doi: 10.3390/ijms26052358 (PMC11900205; doi:10.3390/ijms26052358)
Supplement: Supplementary file 1 [file ijms-26-02358-s001.zip › ijms-3464486-supplementary.pdf]

Tables

Table. S1. Primers for *EsCHS* cloning and expression, chitin synthesis pathway- related genes expression, and RNAi technology

Primer name	Primer sequences (5′→3′)	Product size/bp	Purpose
EsCHS F	AGATAAGTCACCTTGCCACTCG	5236	Gene cloning
EsCHS R	TGGTTAGTCATCAAGGACACGG		
q*EsCHS* F	TGGCAAGTTTGCATGTAAGA	83	qRT-PCR
q*EsCHS* R	GGGATAGGGACACTGGGAT		
qβ-actin F	GCATCCACGAGACCACTTACA	266	
qβ-actin R	CTCCTGCTTGCTGATCCACATC		
q*EsTRE*1-F	GAGGTGAAAACACACAAGAGGC	128	
q*EsTRE*1-R	GTGGAAGGTAGTGACCCAAGAG		
q*EsTRE*2-F	AAAGATACAGCCAGAGATGCCG	125	
q*EsTRE*2-R	CTCAGGACGTCCAAGAGTTACC		
q*EsHK*-F	ACTGCCGTATTGGACTCATTGT	118	
q*EsHK*-R	GTGTTGATGATCACCTGGGGAG		
q*EsG6PI*-F	TCCTCGGACTCCCTCTCAAAAA	117	
q*EsG6PI-R*	GGAGAACAGACAGTGGTGGAAT		
q*EsPAGM*-F	GGGTTATGTGAGGCCGTGAT	110	
q*EsPAGM-R*	AGGGCAGAGGAACTGGACTTT		
q*EsUAP*-F	ATGGACAGCACTTCATCCAAAC	185	
q*EsUAP-R*	AGCTTCTCGAACCCATCAAAGT		
EsCHS-dsRNA F	taatacgactcactatagggGTTCAGGTCTACCCGCATGT	493	RNAi
EsCHS-dsRNA R	taatacgactcactatagggCTTCCACAGCGAGACAAACA		
GFP-dsRNA F	gcgtaatacgactcactataggTGGTCCCAATTCTCGTGGAAC	467	
GFP-dsRNA R	gcgtaatacgactcactataggCTTGAAGTTGACCTTGATGCC		

F and R mean forward primer and reverse primer, minuscule shows T7 Promoter. GFP represents

green fluorescent protein.

Table. S2. Insects and crustacea genes used in the phylogenetic tree analysis of CHS1 and CHS2

Species	GenBank
Macrobrachium nipponense	AKN90966.1
Penaeus monodon	XP_037776976.1
Penaeus vannamei	XP_027227734.1
Portunus trituberculatus	XP_045133051.1
Lepeophtheirus salmonis	MH350852、MH350851
Tigriopus japonicus	AQZ26763.1、AQZ26764.1
Tribolium castaneum	NP_001034492.1、NP_001034491.1
Chilo suppressalis	QJF54125.1
Bactrocera dorsalis	AGC38392.1、AGB51153.1
Locusta migratoria	AFQ00931.1
Sogatella furcifera	ASU54783.1
Nilaparvata lugens	AEL88648.1
Drosophila melanogaster	NP_524209.3
Anthonomus grandis	AHY28560.1
Leptinotarsa decemlineata	LM23646.1
Daphnia magna	KZS18078.1、JAM89352.1
Daphnia pulex	XP_046441590.1

The species name and GenBank number are listed.
